# Mission SpaceX CRS-16 RRRM-1 space flight induced skin genomic plasticity via an epigenetic trigger

**DOI:** 10.1016/j.isci.2024.111382

**Published:** 2024-11-14

**Authors:** Kanhaiya Singh, Priyanka Verma, Rajneesh Srivastava, Yashika Rustagi, Manishekhar Kumar, Sumit S. Verma, Sujit Mohanty, Afshin Beheshti, Liz Warren, Chandan K. Sen

**Affiliations:** 1 Center for Space Biomedicine at McGowan Institute for Regenerative Medicine, Department of Surgery, University of Pittsburgh School of Medicine, Pittsburgh, PA, USA; 2 Indiana Center for Regenerative Medicine and Engineering, Department of Surgery, Indiana University School of Medicine, Indianapolis, IN, USA; 3 Center for the Advancement of Science in Space, Houston, TX, USA

**Keywords:** Histology, space medicine, epigenetics, space sciences

## Abstract

Genomic plasticity helps adapt to extreme environmental conditions. We tested the hypothesis that exposure to space environment (ESE) impacts the epigenome inducing genomic plasticity. Murine skin samples from the Rodent Research Reference Mission-1 were procured from the International Space Station (ISS) National Laboratory. Targeted RNA sequencing to test differential gene expression between the skin of ESE versus ground controls revealed upregulation of VEGF-mediated angiogenesis pathways secondary to promoter hypomethylation in responders. Methylome sequencing identified ESE-sensitive hypomethylated genes including developmental angiogenic genes *Araf*, *Vegfb*, and *Vegfr1*. Based on differentially expressed genes, the angiogenesis biofunction was enriched in responders. The induction of genomic plasticity in response to ESE, as reported herein, may be viewed as a mark of biological resilience that is evident in a minority of organisms, responders but not in non-responders, exposed to the same stressor. Inducible genomic plasticity may be implicated in natural resilience to ESE.

## Highlights

• Exposure to space environment causes genome-wide adaptive epigenetic changes
• Space-exposure adaptive genome-wide changes are only seen in select “responder” mice
• In space, genome-wide epigenetic changes mark induction of genomic plasticity
• Genome-wide hypomethylation in space-exposed mice cause adaptive gene expression

## Graphical abstract

**Figure:**
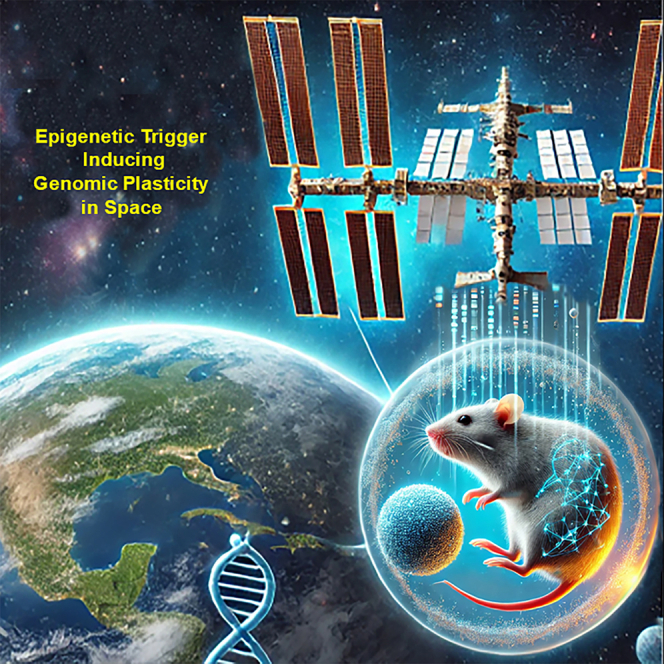

## Introduction

Genomic plasticity, manifested as cell and tissue plasticity, lays the foundation for mammalian life to adapt to extreme environmental condition.^1^ In neo-Darwinian theory, adaptation results from a response to selection on relatively slowly accumulating genetic variation. However, more rapid adaptive responses are possible if selectable or plastic phenotypic variation is produced by epigenetic differences in gene expression.^2^ Epigenetic alterations in the absence of genetic change can affect gene expression.^3^ These epigenetic changes may regulate genome activity independent of DNA sequence and are mitotically stable.^4^ If epigenetic signals producing phenotypic variation are inherited, they can form the basis of adaptive evolutionary change.^5^^,^^6^ Rapid adaptive responses based on epigenetics are possible because the rate of epimutations from DNA methylation has been shown to be orders of magnitude higher than the rate of genetic mutations.^7^ Theoretical investigations have suggested that populations may respond to the environment through epigenetic variation before genetic mutations begin to accumulate. Epigenetic regulation works hand-in-hand with genetic processes to physiologically adapt the organism for the external environment where it will live and reproduce, providing a potential mechanism by which it can cope with ever-changing environmental conditions.^8^ Epigenetic variation such as DNA methylation is found in most species, and variation is common among populations.^2^

The limits of living processes that determine human health, disease, and survival are primarily informed by studies conducted under terrestrial ground conditions. Based on such understanding of physiology on ground conditions, the conceptual limits of biology are currently set. Our current understanding of the limits of regenerative and reparative biology has been thus defined. Studies under extreme environmental conditions such as high altitude and undersea have taught us that genomic plasticity is implicated in enabling adaptations to cope with acclimatization.^9^ Genomic plasticity translates to tissue plasticity and is manifested as functional adaptations, thus stretching the biological limits compared to what is known under standard ground conditions. The most rapid-response component of genomic plasticity is represented by epigenetic changes. During the reprogramming process, to acquire plasticity, cells reset their pattern of DNA methylation and post-translational histone modifications and lose their “epigenetic memory.”^10^ Interestingly, such epigenetic changes in individuals are more proactive in space.^11^ DNA methylation is one of such essential epigenetic mechanisms that actively regulates gene expression and maintains genomic integrity. In the current work, we test the hypothesis that exposure to space environment (ESE) profoundly impacts the epigenome as marked by DNA hypomethylation to induce rodent genomic plasticity in responders. Non-responders fail to respond to adaptive cues elicited by ESE.

## Results

### ESE induces stemness in the murine skin

**Figure 1:**
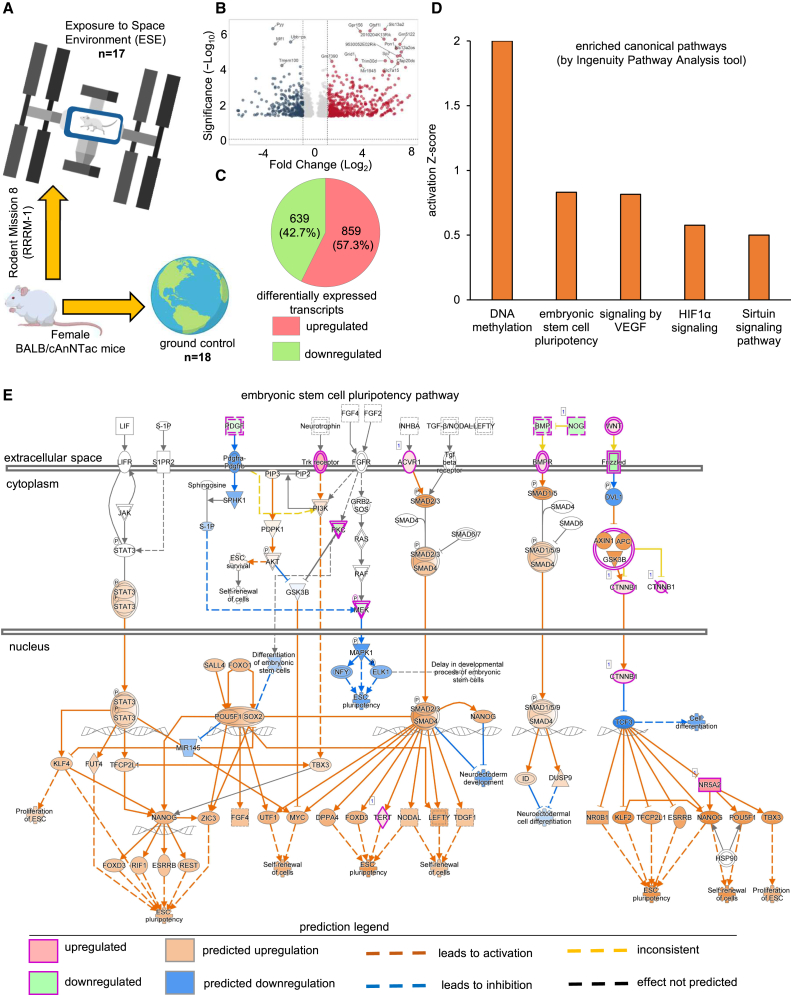
ESE induces stemness in the murine skin (A) Total study samples: mice skin samples from the Rodent Research-8 mission (also known as Rodent Research Reference-1) were procured from International Space Station (ISS) National. Mice flown in animal enclosure modules on the ISS were classified as Exposure to Space Environment (ESE) group (*n* = 17). Ground-based mice (ground controls; *n* = 18) maintained under similar housing conditions were used as controls. Image created in BioRender. Kanhaiya, K. (2024) https://BioRender.com/q43i834. (B) To quantify transcriptional differences between the skin of the mice of ESE group (*n* = 10) vs. the ground controls (*n* = 6), targeted RNA sequencing was performed. A total of 1,498 analysis ready differentially expressed transcripts (*p* value < 0.05) were identified using ingenuity pathway analysis (IPA) software. Volcano plot and (C) Venn diagram showing the comparison of differentially expressed genes (DEG) in ESE group skin sample 859 (57.3%) transcripts were upregulated while 639 (42.7%) were downregulated. (D) Canonical pathway enrichment analysis of DEGs, high in ESE group was performed using IPA. Presented canonical pathways were identified as (1) DNA methylation, (2) embryonic stem cell pluripotency, (3) signaling by VEGF, (4) HIF1α signaling, and (5) Sirtuin signaling pathway. (E) Pathway generated by IPA showing the candidate genes involved in the human embryonic stem cell (ESC) pluripotency pathway. Among the biofunctions that were predicted to be upregulated in the skin samples of ESE group were ESC pluripotency, self-renewal of cells, and proliferation of ESC. See also Figure S5 and Tables S1 and S2; n represents number of animals. For a Figure360 author presentation of this figure, see https://doi.org/10.1016/j.isci.2024.111382.

Mice skin samples from the Rodent Research-8 mission (also known as Rodent Research Reference Mission-1) were procured from International Space Station (ISS) National Laboratory. Mice were flown in an established Rodent Habitat^12^ on the ISS (*n* = 17) (Figure 1A). Ground-based mice (ground controls; *n* = 18) maintained under similar housing conditions were used as controls (Figure 1A). Differentially expressed genes (using DE-seq2) followed by identification of altered pathway between the groups was performed using Ingenuity Pathway Analysis (IPA) software. A total of 1,498 analysis ready differentially expressed transcripts (*p* value<0.05) were identified (Figures 1B and 1C; Table S1). Comparison of differentially expressed transcripts in the skin sample from ESE group identified that 859 (57.3%) transcripts were upregulated, whereas 639 (42.7%) were downregulated (Figure 1C). Canonical pathway enrichment analysis of DEGs, high in ESE group, was performed using IPA (Figure 1D). The presented canonical pathways were identified as (1) DNA methylation, (2) embryonic stem cell (ESC) pluripotency, (3) signaling by VEGF, (4) hypoxia-inducible factor 1α (HIF1α) signaling, and (5) Sirtuin signaling pathways (Figure 1D). Among the genes of the ESC pluripotency pathway that were upregulated include activin A receptor type 1 (*Acvr1*), β-catenin 1 (*Ctnnb1*), nuclear receptor subfamily 5 group A member 2 (*Nr5a2*), neurotrophic receptor tyrosine kinase 3 (*Ntrk3*), protein kinase D1 (*Prkd1*), telomerase reverse transcriptase (*Tert*), and Wnt family member 6 (*Wnt6*) (Table S2). Biological functions that were predicted to be upregulated based on DEGs were ESC pluripotency, self-renewal of cells, and proliferation of ESC (Figure 1E).

### Dichotomous 5-methylcytosine levels in ESE: Responders and non-responders

**Figure 2:**
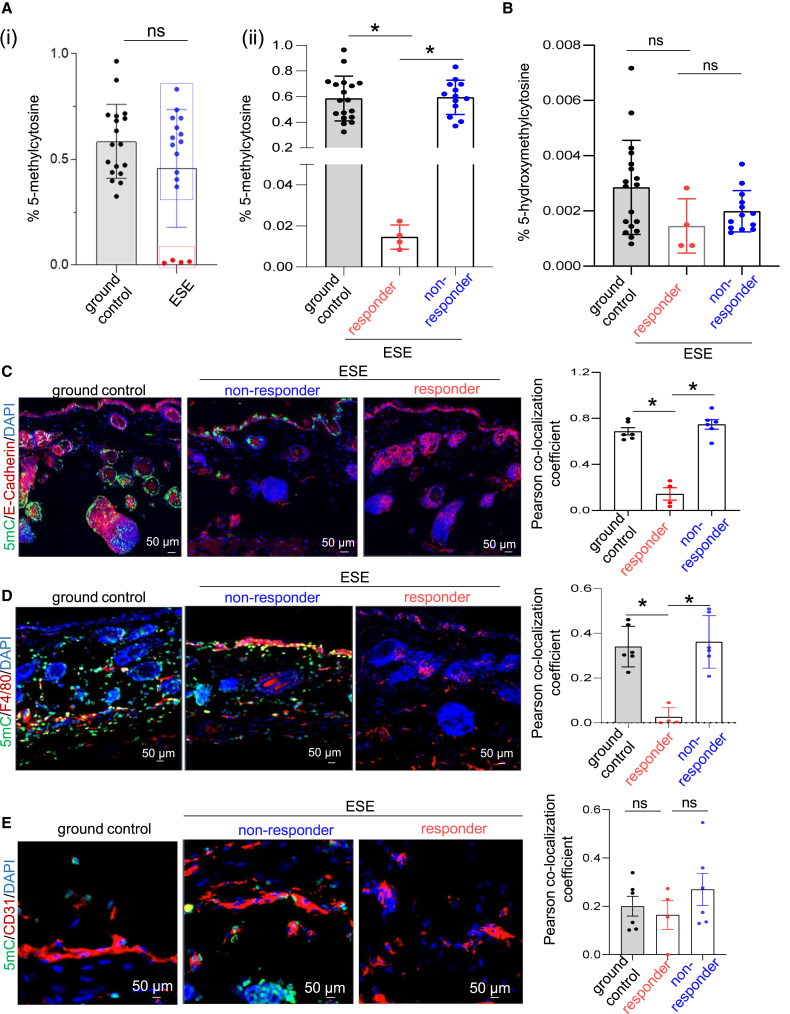
Dichotomous 5-methylcytosine levels in ESE responders and non-responders (A) The percentage of 5-methylcytosine (5mC) as measured using an enzyme-linked immunosorbent assay (ELISA) kit in (i) ground control (*n* = 18) and ESE (*n* = 17) groups; (ii) the ESE groups were further divided into responder ESE cohort (*n* = 4) and non-responder ESE cohort (*n* = 13) based on 5mC percentage. Data presented as mean ± SD. ∗*p* < 0.05; one-way ANOVA followed by Tukey’s multiple comparisons test. (B) The percentage of 5-hydroxymethylcytosine (5hmC) as measured using an ELISA kit in ground control and aforementioned responder ESE cohort (*n* = 4) and non-responder ESE cohort (*n* = 13). Data presented as mean ± SD. (C) Immunohistochemical (IHC) analysis of 5mC+/E-cadherin+ colocalization in ground control (*n* = 6), responder ESE cohort (*n* = 4), and non-responder ESE cohort (*n* = 6). Colocalization coefficients are represented as the mean ± SEM. ∗*p* < 0.05; one-way ANOVA followed by Tukey’s multiple comparisons test. (D) IHC analysis of 5mC+/F4/80+ colocalization in ground control (*n* = 6), responder ESE cohort (*n* = 4) and non-responder ESE cohort (*n* = 6). Colocalization coefficients are represented as the mean ± SEM. ∗*p* < 0.05; one-way ANOVA followed by Tukey’s multiple comparisons test. Scale bars, 50 μm. (E) IHC analysis of 5mC+/CD31+ colocalization in ground control (*n* = 6), ESE responders (*n* = 4), and ESE non-responders (*n* = 6). Colocalization coefficients are represented as the mean ± SEM. Scale bars, 50 μm. See also Table S3. n represents number of animals.

IPA analyses of differentially expressed transcripts identified DNA methylation as the primary canonical pathway to be significantly enriched in murine skin post-ESE (Figure 1C). Follow-up studies on DNA methylation was therefore performed. These studies recognized that responses to the exposure to ESE is dichotomous, thus identifying two distinct groups based on global methylation levels—non-responders and responders. In responders, but not in non-responders, 5-methylcytosine (5-mC; marker of DNA methylation) levels were sharply low (Figures 2A and 2B; Table S3). Conventionally, lowering of 5-mC denotes demethylated DNA associated with induction of corresponding gene expression.^13^^,^^14^ Immunohistochemical studies demonstrated that the ESE-responsive 5-mC-lowering effect was confined to specific E-cadherin^+^ epithelial and F4/80^+^ myeloid cell compartments (Figures 2C and 2D). Endothelial (PECAM1^+^) compartment was not involved in such kind of epigenetic regulation (Figure 2E). To understand the effect of the observed global methylation changes on specific genes and related pathways, an unbiased DNA methylation (methylome) sequencing approach was adopted (Table S4).

### ESE induced genome-wide hypomethylation in responders

**Figure 3:**
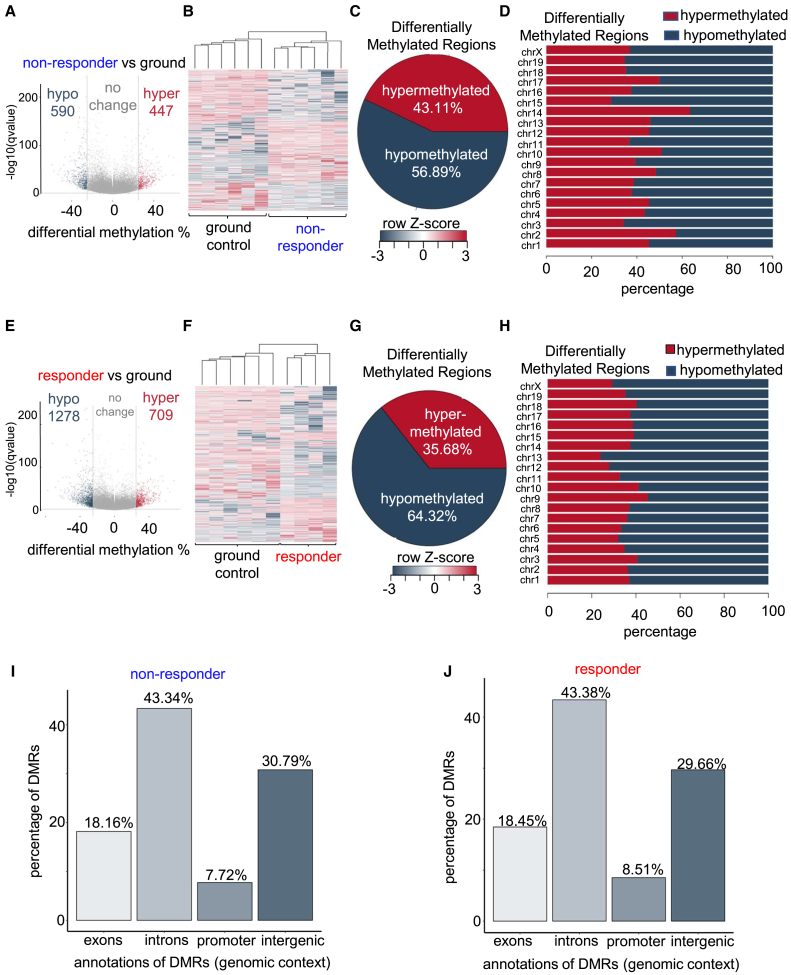
ESE induced genome-wide hypomethylation in responders (A) Volcano plot of total methylated regions in non-responder ESE cohort (*n* = 6) compared to ground controls (*n* = 6). Out of total 79,487 identified differentially methylated regions (DMRs), 1,037 significant DMRs (differentially methylated with a q-value smaller than 0.01 and at least 25% difference) were identified. Out of 1,037 significant DMRs, 590 were hypomethylated in responder ESE cohort and 447 were hypermethylated. (B) Clustering heatmap of significant DMRs identified in (A) and a heatmap was generated. (C) The pie chart shows the percentage of hyper- and hypomethylated regions in the comparison. Out of 1,037 significant DMRs, 590 (56.89%) were hypomethylated in responder ESE cohort and 447 (43.11%) were hypermethylated. (D) The percentage of hypo- and hypermethylated regions is plotted in a bar chart per chromosome in non-responder ESE cohort compared to ground controls. (E) Volcano plot of DMRs in responder ESE cohort (*n* = 4) compared to ground controls (*n* = 6). Out of total 82,489 identified DMRs, 1,987 significant DMRs (differentially methylated with a q-value smaller than 0.01 and at least 25% difference) were identified. Out of 1,987 significant DMRs, 1,278 were hypomethylated in responder ESE cohort and 709 were hypermethylated. (F) Clustering heatmap of significant DMRs in responder ESE cohort (*n* = 4) compared to ground controls (*n* = 6). The 1,987 significant DMRs (differentially methylated with a q-value smaller than 0.01 and at least 25% difference) were clustered, and a heatmap was generated. (G) The pie chart shows the percentage of hyper- and hypomethylated regions in the comparison. Out of 1,987 significant DMRs, 1,278 (64.32%) were hypomethylated in responder ESE cohort and 709 (35.68%) were hypermethylated. (H) The percentage of hypo- and hypermethylated regions is plotted in a bar chart per chromosome in responder ESE cohort compared to ground controls. (I) Bar graph showing the percentage genomic distribution (exons, introns, promoters, or intergenic) of DMRs in non-responder and (J) in responder ESE groups. See also Figures S1 and S2, Table S4. n represents number of animals.

**Figure 4:**
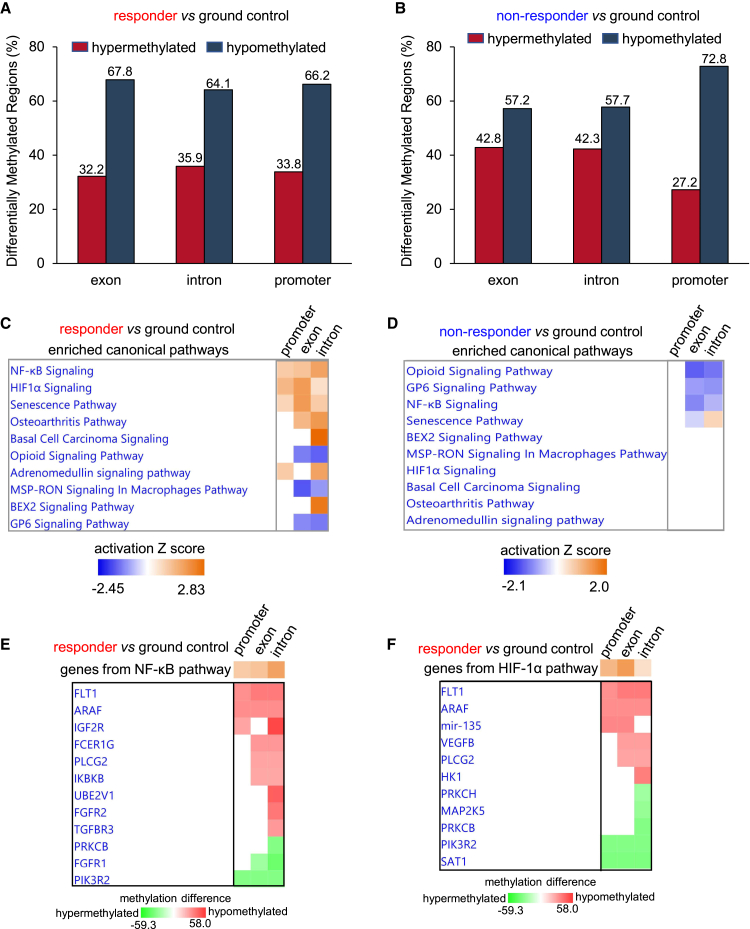
ESE-induced promoter hypomethylation increased angiogenic gene expression (A) Bar graph showing significant DMRs annotated in exon, intron, and promoter regions in responder ESE cohort (*n* = 4) compared to ground controls (*n* = 6). (B) Bar graph showing significant DMRs annotated in exon, intron, and promoter regions in non-responder ESE cohort (*n* = 6) compared to ground controls (*n* = 6). (C) Canonical pathways enriched by the DMRs annotated in exon, intron, and promoter regions in responder ESE cohort (*n* = 4) compared to ground controls (*n* = 6) using comparison analysis function of ingenuity pathway analysis (IPA). (D) IPA generated canonical pathways in non-responder ESE cohort (*n* = 6) enriched by the DMRs annotated in exon, intron, and promoter regions as compared to ground controls (*n* = 6). (E) Heatmap showing the expression pattern of genes involved in NF-κB pathway and (F) HIF1α pathway in responder ESE cohort (*n* = 4) as compared to ground controls (*n* = 6). n represents number of animals.

Pairwise comparison using reduced representation bisulfite sequencing (RRBS) identified statistically significant DMRs with a q-value cutoff <0.01 and a methylation difference higher than 25%. The number of hypomethylated DMRs in responder ESE cohort (1278) was >2-fold high compared to non-responder ESE cohort (590) with respect to the ground controls (q-value cutoff <0.01 and a methylation difference >25%) (Figures 3A–3H). Such increased number of hypomethylated DMRs were also observed in responders when directly compared with non-responders (Figures S1A–S1C). Interestingly, such differential methylation events were not specific to any particular genomic region or chromosome between responder and non-responder ESE cohort indicative of a global demethylation process (Figures 3I, 3J, and S1D). IPA of differential genetic pathways in responders identified the top three canonical pathways that were significantly hypomethylated. Nuclear factor κB (NF-κB), HIF-1α, and senescence pathways were hypomethylated in responders compared to ground controls or the non-responder ESE cohort (Figures 4A–4D and S1E). Identification of candidate genes enriched in the aforementioned pathways revealed that the developmental angiogenic genes such as FMS-like tyrosine kinase 1 (*Flt1*, also known as vascular endothelial growth factor receptor [*Vegf-R1*]), serine/threonine-protein kinase A-Raf *(Araf)*, and *Vegfb* were hypomethylated in the responder ESE cohort (Figures 4E and 4F). Such hypomethylation of candidate genes has been known to cause adaptive changes (e.g., angiogenesis) under terrestrial conditions.^15^^,^^16^^,^^17^ On the contrary, promoter hypermethylation of *Flt1* correlates with decreased expression and function of VEGF pathway genes impairing vascularization.^18^^,^^19^ To further understand the functional effects of such hypomethylation on gene expression, the DEGs between the non-responders and responders were analyzed using disease and function analysis tool in IPA software. Non-responder mice were associated with loss of muscle function and contractility, loss of developmental process of synapse, diminished fatty acid metabolism, and gain of myopathy function as compared to responders (Figure S2). Responders were more resilient. Follow-up immunohistochemical studies validated that the expression of the corresponding proteins was indeed upregulated in the tissue of the responders.

### ESE-induced promoter-hypomethylation increased angiogenic gene expression

**Figure 5:**
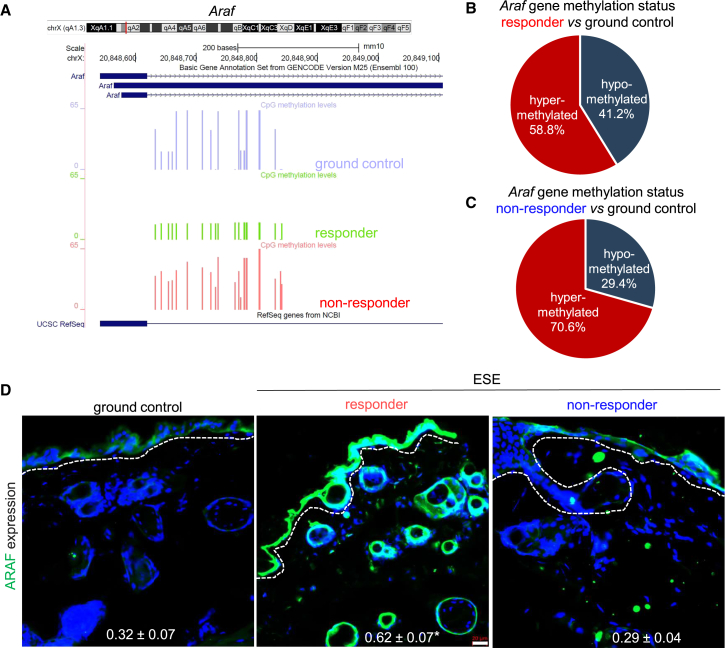
ESE-induced gene hypomethylation increased angiogenic gene expression (A) Targeted validation of *Araf* gene methylation status in ground control, responder ESE cohort, and non-responder ESE cohort. Genome track showing the *Araf* locus (top). Representative DNA methylation tracks (bottom) show diminished levels of methylated CpGs in responder ESE skin as compared to non-responder ESE or control skin. Full image of genome track is also shown in Figure S3. (B) Venn diagram showing the percentage of hypomethylated (blue) and hypermethylated (red) CpGs in *Araf* gene in responder ESE cohort (*n* = 4) vs. ground control (*n* = 12) and (C) in non-responder ESE cohort (*n* = 6) vs. ground control (*n* = 12). (D) IHC analysis of ARAF intensity in ground control, ESE responders, and ESE non-responders (*n* = 3). Colocalization coefficients are represented as the mean ± SEM on the image. ∗*p* < 0.05; responder ESE vs. ground control; one-way ANOVA followed by Tukey’s multiple comparisons test. Scale bar, 20 μm. n represents number of animals.

**Figure 6:**
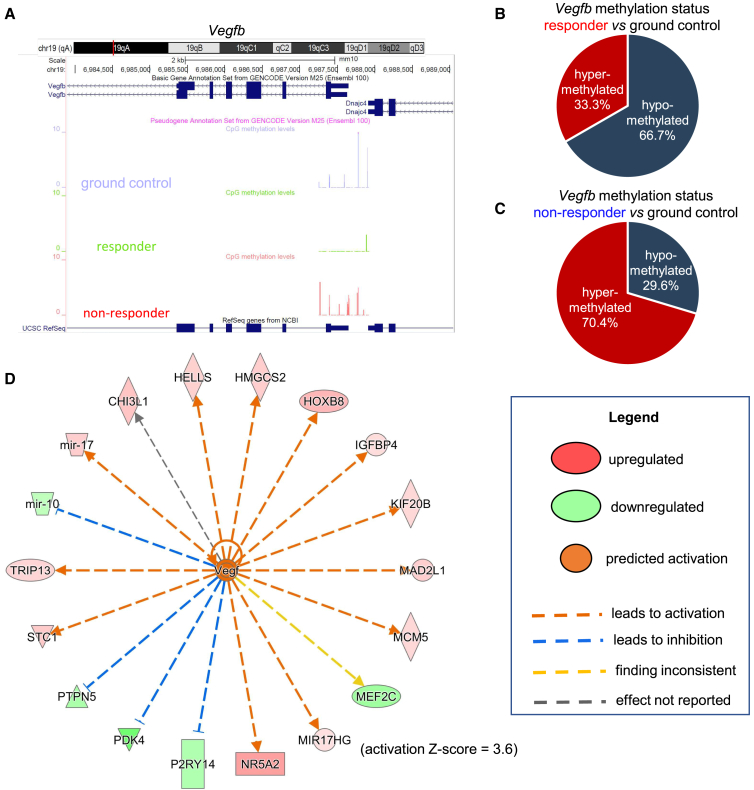
ESE-induced promoter hypomethylation increased VEGF-mediated angiogenesis pathway in responders (A) Targeted validation of *Vegfb* gene promoter methylation status in ground control, responder ESE cohort, and non-responder ESE cohort. Genome track showing the *Vegfb* locus (top). Representative DNA methylation tracks (bottom) show diminished levels of methylated CpGs in responder ESE skin as compared to non-responder ESE or control skin. (B) Venn diagram showing the percentage of hypomethylated (blue) and hypermethylated (red) CpGs in *Vegfb* gene in responder ESE cohort (*n* = 4) vs. ground control (*n* = 12) and in (C) non-responder ESE cohort (*n* = 6) vs. ground control (*n* = 12). (D) To quantify transcriptional differences between the skin of the mice of responder ESE cohort compared to ground controls, targeted RNA sequencing was performed. Upstream regulator prediction analysis of DEGs, high in responder ESE cohort was performed using IPA. The candidates from VEGF Pathway were enriched in responder ESE cohort. See also Figure S4, Table S5. n represents number of animals.

Global RRBS methylome analysis revealed that developmental angiogenesis markers such as *Flt1* and *Araf* were hypomethylated in all three major genomic compartments, i.e., promoter, exon, and intron. ESE-induced significant hypomethylation was observed in the *Araf* gene in the responder ESE cohort (41.2%) vs*.* non-responder cohort (29.4%) when compared individually to ground controls (Figures 5A–5C and S3). Such hypomethylation was associated with an increase in the protein expression of ARAF in the responder ESE cohort (Figure 5D). A similar trend of hypomethylation was observed in other key angiogenic genes *Flt1* and *Vegfb* when investigated using targeted bisulfite sequencing (Figures 6A–6C and S4A–S4C). Based on DEGs, angiogenesis biofunction was enriched in responders (activation *Z* score = 1.8) compared to non-responders (activation *Z* score = 0.7). The analyses of upstream regulators predicted the activation of VEGF in responders (activation *Z* score = 3.6) (Figure 6D) compared to non-responders (activation *Z* score = 2.7). Taken together, these two sets of high-throughput genomic analyses (RRBS and mRNAseq) demonstrated the upregulation of VEGF-mediated angiogenesis pathway and found to be activated in responder ESE cohort.

## Discussion

In the timescale of 3.8 billion years of the existence of earth, bipedal hominins have evolved over a span of seven million years.^20^ Rodents have existed on earth for an estimated 100 million years.^21^ It is during this time span that evolution on earth has produced phenotypes adjusted to life on earth. Developmental plasticity, adaptive and responsive to natural selection, has made distinct phenotype choices from the same genotype resources. Evolutionary and ecological forces have determined the phenotype of taxa. If this is the basis of what we today know as terrestrial physiology, the question is how rapidly life may re-adjust in response to exposure to extreme living conditions, terrestrial or extra-terrestrial. To life forms adjusted to terrestrial living, space presents itself as a hostile environment.^22^ To cope with extra-terrestrial conditions, terrestrial living organisms must undergo substantial adaptations in the respiratory and metabolic processes, muscle and bone composition, and body mass.^23^ In humans, such adaptation to ESE involves an array of genomic and epigenomic changes.^23^ In addition to such short-term effects, ESE has the clear potential to impact evolutionary biology.^23^ The ability of the body to be responsive to such demands of survival is a measure of resilience.

Current research in space biology predominantly focuses on the loss of bodily structure and function with respect to terrestrial living. Specific examples include loss of bone and muscle mass, deconditioning of the cardiovascular system, immune-dysregulation, and changes in metabolism. Although these changes are broadly categorized as “maladaptation,” it must be recognized that such changes represent a partial view of bodily adaptations as it seeks to position itself to survive in a spaceship under ESE conditions. Rarely are we interested in any gain of bodily function that is likely to be in play concurrent with the aforementioned “maladaptation” as the body seeks to cope and survive under extra-terrestrial conditions. To appreciate the significance of resilient changes occurring in the body under extra-terrestrial conditions, we must adopt a perspective that is different from the assumption that any departure from the norms of terrestrial living is maladaptive and pathological. The induction of genomic plasticity in response to ESE, as reported herein, may be viewed as a mark of biological resilience that is evident in a minority of organisms exposed to the same stressor.

Exposure to ESE is known to result in biological outcomes that vary from individual to individual. For example, loss of bone or body mass loss varies among individuals.^23^ The ability of the same genotype to produce different phenotypes under different environmental conditions is a notion that is generally accepted in evolutionary biology. A reaction norm defines the range of phenotypes expressed by a genotype along an environmental gradient.^24^ The ESE responder-non-responder paradigm that has emerged in this work supports the notion that the sensitivity of genomic plasticity to ESE may be highly contrasting among inbred mice of comparable genotype. From a pool of over 12,000 people who applied to be an astronaut, NASA selected 10 as astronaut candidates and went on to be flight-eligible. The efficacy of such selection process to identify those who naturally induce genomic plasticity in response to ESE remains to be known. Furthermore, the significance of such natural resilience to ESE for long-term space missions such as the *Human to Mars* mission would be of critical value to select astronauts for such programs.

The NASA twins study report on epigenetic changes in response to actual or simulated ESE.^25^ In the CD4 cell compartment under inflight conditions, subtle decrease in genome-wide mean methylation levels was reported. The comparison of local methylation changes in inflight compared to preflight revealed the enrichments for genes involved in the response to platelet-derived growth factor (PDGF) pathway. In addition, the plasma levels of VEGF increased in the astronaut exposed to ESE in the days after return and continued to be increased 6 months after return. Taken together, ESE demethylated and thus enhanced the expression of angiogenic genes. Such findings are in concordance with the overall findings of this work. The vascular system appears to be particularly responsive to ESE.^26^^,^^27^ Blood redistribution is widely acknowledged as an acute phase response to microgravity.^28^ The terrestrial paradigm of highest arterial pressure in the feet and lowest in the head is upset in microgravity, thus placing a demand on the vascular system to cope.^22^ Functional adaptation of the vascular system to microgravity includes upregulation of nitric oxide synthase (NOS) and subsequent nitric oxide (NO) synthesis through activation of PI3K-Akt-eNOS signaling pathway.^29^ These changes in gene expression can be attributed to the combined loss of abundance of the DNA methyltransferase family members (DNMT1, 3a, and 3b) and gain in the levels of ten-eleven translocation (TET) family proteins (TET1, 2 and 3) following long-term exposure to ESE, which in turn induces promoter hypomethylation.^30^^,^^31^^,^^32^ In this work, hypomethylation-induced increase in *Vegf* and *Flt1* gene expression was observed in responder ESE cohort. These genes are known to signal via the PI3K-Akt-eNOS pathway.^33^

This is one of the first reports on mission RRRM-1 providing the context for future literature on this mission. Because the data presented in this paper are from females, sex-specific differences are not captured. Certain DNA methylation patterns and the levels of methyl transferases along with other epigenetic regulators may vary with sex.^34^^,^^35^ To address this limitation, additional analysis on the femoral skin from male mice from JAXA MHU-2 mission was conducted using NASA Open Science for Life in Space repository (GeneLab ID: GLDS-239; https://doi.org/10.26030/s7k9-7958). Upregulation of predicted biological functions such as ESC pluripotency, self-renewal of cells, and proliferation of ESC was also observed in male mice similar to females (Figure S5A). The other pathways that followed the similar upregulation in males upon ESE were signaling by VEGF, HIF signaling, and sirtuin signaling pathway (Figure S5B). These observations lay the foundation to future studies addressing this paradigm in humans.

The larger goal of understanding genomic plasticity as a function of ESE is likely to define limits for mammalian adaptive physiology. Thus, a paradigm would emerge wherein our understanding of mammalian physiology and its ability to cope with stress will be transformed. This would set the stage for teasing out specific variables, components of ESE, that induce rapid epigenetic changes and translating the knowledge gained about tissue plasticity to develop therapeutic strategies. On the scientific front, we are likely to find cell states and fates that would be responsible for setting functional limits of the body. Future studies will employ tools such as single-cell RNA sequencing and spatial transcriptomic studies to gain fundamental insights in tissue and organ biology. On the translational front, regenerative therapies will be inspired by the findings of the proposed work.

### Limitations of the study

Limitations of this study include a relatively small sample size, and the obvious challenges associated with collection and return of in-flight samples^36^^,^^37^ contributing factors such as microgravity, space radiation exposure, psychological stress, nutrition, and exercise cannot be isolated and tested, so the ESE studied in this work involves numerous variables in play ranging from known factors such as microgravity to ESE factors that remain to be identified. For the purposes of this work, it was not of interest to isolate the variables in play and therefore we consider ESE as one combined variable.

## Resource availability

### Lead contact

Further information and requests for resources and reagents should be directed to and will be fulfilled by the lead contact, Chandan K Sen (c.k.sen@pitt.edu).

### Materials availability

This study did not generate new unique reagents.

### Data and code availability

Generated bulk RNA-seq and RRBS methylation data have been deposited at GEO under accession numbers GSE268753 and GSE268755. This paper does not report any original code. Any additional information required to analyze the data reported in this paper is available from the lead contact upon request.

## Acknowledgments

Research programs of K.S. is supported by 10.13039/100000005U.S. Department of Defense grants W81XWH-22-1-0146 & HT94252410131; NIH-R01 grant DK136814-01; and grants from the Commonwealth of Pennsylvania. Research programs of C.K.S. is supported by NIH-R01 grants DK128845, DK141513, DK135447, and DK125835.

## Author contributions

K.S., P.V., and C.K.S. designed the study. K.S., P.V., Y.R., M.K., S.V., S.M., and R.S. participated in experiments and related data analysis. K.S. and C.K.S. wrote the manuscript. L.W. and A.B. reviewed and edited the manuscript. All authors read and approved the final manuscript.

## Declaration of interests

The authors declare no competing interests.

## Declaration of generative AI and AI-assisted technologies in the writing process

During the preparation of this work, the authors used Dall E3 to generate the schematics for graphical abstract. After using this tool, the authors reviewed and edited the content using Adobe Photoshop and take full responsibility for the content of the publication.

## STAR★Methods

### Key resources table

**Table:** 

REAGENT or RESOURCE	SOURCE	IDENTIFIER
**Antibodies**		

anti-5-mC (rabbit monoclonal)	Cell Signaling Technology	Cat# 28692S; RRID: AB_2798962
anti-5-hmC (rabbit monoclonal)	Abcam	Cat# ab214728; RRID: AB_2797407
anti-E-cadherin (rat monoclonal)	Invitrogen	Cat# 13–1900; RRID: AB_2533005
anti-CD31 (rat monoclonal)	BD Pharmingen	Cat# 550274; RRID: AB_393571
anti-F4/80 (rat monoclonal)	Bio-Rad	Cat# MCA497R; RRID: AB_323279
anti-ARAF (rabbit polyclonal)	Proteintech Group	Cat# 22129-1-AP; RRID: AB_2879001
Alexa 568-tagged α-sheep	Invitrogen	Cat# A-21099; RRID: AB_2535753
Alexa 568-tagged α-rat	Abcam	Cat# ab175476; RRID: AB_2813739
Alexa 488-tagged α-rabbit	Invitrogen	Cat# A-11008; RRID: AB_143165

**Biological samples**		

Femoral skin samples (∼10–16 weeks) female BALB/cAnNTac mice, Taconic Biosciences	International Space Station (ISS) National Laboratory’s Biospecimen Sharing Program	Rodent Research-8 mission (RRRM-1)

**Critical commercial assays**		

DNeasy Blood & Tissue Kit	Qiagen	69504
MethylFlash™ Methylated DNA Quantification ELISA Kit	Epigentek	P-1030
MethylFlash™ Global DNA Hydroxymethylation (5-hmC) ELISA Kit	Epigentek	P-1032
PicoPure™ RNA Isolation Kit	Applied Biosystems	KIT0204
RNase Cocktail	Invitrogen	AM2288
Agencourt® AmpureXP beads	Beckman Coulter	A63882
DNF-488 High Sensitivity genomic DNA Analysis Kit	Agilent	DNF-488-FR
Premium Reduced Representation Bisulfite Sequencing (RRBS) Kit	Diagenode	C02030033
EZ DNA Methylation-Lightning™ Kit	Zymo Research	D5030
MiSeq V2 300bp Reagent Kit	Illumina	MS-102-2001

**Deposited data**		

Raw and analyzed data	This paper	GEO: GSE268753 and GSE268755
Raw and analyzed data	JAXA MHU-2 mission	GeneLab ID: GLDS-239; https://doi.org/10.26030/s7k9-7958

**Software and algorithms**		

ImageJ	NIH	https://imagej.nih.gov/ij/
Zen software	Zeiss microscopy	https://www.zeiss.com/microscopy/en/products/software/zeiss-zen-lite.html
FastQC/MultiQC	N/A	0.11.5
STAR	Dobin et al.^38^	2.5
NGSUtils, bamutils	Breese et al.^39^	0.5.9
subread/featureCounts	Liao et al.^40^	1.5.1
edgeR	Robinson et al.^41^	3.30.3
Refernce genome	UCSC	mm10/refGene
Methylkit	R/Bioconductor	1.7.0
annotatr	R/Bioconductor	NA
Hierarchical Indexing for the Spliced Alignment of Transcripts (HISAT2)	Kim et al.^42^	NA
SAMtools	Li et al.^43^	0.1.19
Ingenuity Pathway Analysis (IPA) database	QIAGEN Inc	NA
StringTie	Pertea et al.^44^	1.2.1
DE-seq2	Love et al.^45^	NA
ggplot2	Ito et al.^46^	NA

**Other**		

Zeiss Axio Scan fluorescence microscope	Zeiss microscopy	https://www.zeiss.com/microscopy/en/products/imaging-systems/axioscan-for-biology.html
Bioanalyzer 2100	Agilent	https://www.agilent.com/en/product/automated-electrophoresis/bioanalyzer-systems/bioanalyzer-instrument
NovaSeq 6000	Illumina	https://www.illumina.com/systems/sequencing-platforms/novaseq.html
4200 TapeStation system	Agilent	https://www.agilent.com/en/product/automated-electrophoresis/tapestation-systems/tapestation-instruments/4200-tapestation-system-228263

### Experimental model and study participant details

#### Skin samples from mice

Femoral skin samples from 35 young (∼10-16 weeks) female BALB/cAnNTac mice (Taconic Biosciences) were obtained through the International Space Station (ISS) National Laboratory’s Biospecimen Sharing Program, and no live animals were involved in this study. These mice belonged to the SpaceX CRS-16 Rodent Research-8 mission [also known as Rodent Research Reference Mission-1 (RRRM-1)] and were procured from ISS National Laboratory in collaboration with Dr. Liz Warren. The detailed information regarding the RRRM-1 mission is available through ISS laboratory’s Center for the Advancement of Science in Space (CASIS) research archive (www.iss-casis.org/research-on-the-iss/solicitations/2018-rodent-research/). Briefly, this study investigated skin samples from 17 mice that were flown to space [10 euthanized on-orbit after 22-23 days; 7 euthanized post-landing (40 days in space and allowed to recover for 2 days before sacrifice) and 18 mice age-matched ground control animals.

### Method details

#### RNA sequencing and analysis

For targeted RNA sequencing, total RNA was extracted using ABI Picopure RNA isolation kit and quality assessment was performed using Agilent 2100 Bioanalyzer. The paired-end library was prepared with 30-200ng of RNA using Agilent SureSelect Custom cDNA Conversion Module, plus SSEL XTHS reagent Kit with Mouse All Exon V2 (67.1Mb). Sequencing was performed using NovaSeq 6000 S2 kit on Illumina NovaSeq 6000 platform with a read length of 100bp. The sequence reads were mapped to the designated reference genome using STAR (Spliced Transcripts Alignment to a Reference).^38^ To evaluate the quality of the RNA-seq data, the number of reads that fall into different annotated regions (exonic, intronic, splicing junction, intergenic, promoter, UTR, etc.) of the reference genes were determined with bamUtils.^39^

For differential gene expression (DEG) analysis, low quality mapped reads (including reads mapped to multiple positions) were excluded and featureCounts^40^ was used to quantify the gene level expression. DEG analysis was performed with edgeR.^41^ In this workflow, the statistical methodology applied uses negative binomial generalized linear models with likelihood ratio tests.

For comparison between ground control and ESE as mentioned in Figure 1, the processed raw sequencing reads were aligned onto the mouse reference genome (mm39) using Hierarchical Indexing for the Spliced Alignment of Transcripts (HISAT2).^42^ Resulting SAM (Sequence Alignment/Map) files were postprocessed using SAMtools (version 0.1.19)^43^ for converting SAM to BAM (Binary Alignment/Map), followed by sorting and indexing the output BAMs. The sorted BAM files were parsed using the python script (prepDE.py) provided by StringTie (version 1.2.1)^44^ to obtain the count matrix for gene levels across the samples. This count matrix was used to perform the differential expression analysis between ESE and ground control samples using DE-seq2.^45^ Differentially expressed and statistically significant genes identified in all comparisons were illustrated in volcano plots generated using ggplot2^46^ package in R.

#### Reduced representation bisulfite sequencing (RRBS) analysis

RRBS analysis was performed using Diagenode Epigenomics Services (Diagenode, Cat# G02020000) on total DNA isolated from murine skin samples using the DNeasy Blood & Tissue Kit (Qiagen, Cat# 69504) as per manufacturer’s instructions. The Quantity and sample integrity assessment of DNA starting material were determined via 4200 TapeStation system (Agilent). Genomic DNA was treated with RNase Cocktail (Invitrogen, Cat# AM2288) for 30min at 37°C to remove contaminating small RNAs. Following the treatment, the DNA was purified using a 2x beads:sample ratio of Agencourt® AmpureXP beads (Beckman Coulter). DNA concentration was measured using the Qubit® dsDNA BR Assay Kit (Thermo Fisher Scientific) and quality was assessed with the Fragment Analyzer™ and the DNF-488 High Sensitivity genomic DNA Analysis Kit (Agilent). RRBS libraries were prepared using the Premium Reduced Representation Bisulfite Sequencing (RRBS) Kit (Diagenode Cat# C02030033). 100ng of genomic DNA were used to start library preparation. Following library preparation, samples were pooled together. PCR clean-up after the final library amplification was performed using a 1.45x beads:sample ratio of Agencourt® AMPure® XP (Beckman Coulter). RRBS library pools were sequenced on a NovaSeq6000 (Illumina) using 50 bp paired-end read sequencing (PE50).

Quality control of sequencing reads obtained was performed using FastQC version 0.11.8. Adapter removal was perfomed using Trim Galore!^47^ version 0.4.1. Reads were then aligned to the mouse reference genome GRCm38/mm10 using bismark v0.20.0.^48^ The spike-in control sequences were used at this step to check the bisulfite conversion rates and to validate the efficiency of the bisulfite treatment. Methylkit v1.7.0,^48^ a R/Bioconductor package, was used to perform the differential methylation analysis. The CpG data set was filtered for low coverage (CpGs with coverage less than 10X in all samples per comparative group were discarded) and for extremely high coverage to discard reads with possible PCR bias (CpGs with coverage higher than the 99.9th percentile were discarded). The data was then normalized for read coverage distribution between samples. A pairwise comparison was performed between groups to identify differentially methylated regions (DMRs), with a window and step size of 1000bp using logistic regression. After the p-values have been computed, methylkit uses the sliding window model (SLIM) to correct P-values to q-values for multiple comparison tests. Statistically significant DMRs were identified with a q-value cutoff < 0.01 and a methylation difference higher than 25%. All DMRs were annotated with the R/Bioconductor package annotatr,^49^ with the refGene and CpG island annotations from UCSC. The annotation comprised two categories: (i) distance to a CpG island and (ii) regional annotation. The regional analysis classified DMRs in three groups, namely, exons, introns and promoters.

#### Ingenuity Pathway Analysis (IPA)

After the identification of DEGs/DMRs, enriched pathways were identified using the Ingenuity Pathway Analysis (IPA) database (QIAGEN Inc., Redwood City, CA, www.qiagen.com/ingenuity) that predicts biological networks enriched in the dataset based on the existing literature. Significant DEGs/DMRs were subjected to canonical pathway analysis using IPA as reported by us previously.^50^^,^^51^^,^^52^ To compare the similarity and difference among the enriched canonical pathways, the comparison analysis was performed using the comparison analysis function in IPA between DMRs corresponding to gene promoter, exons, and introns.^53^ The bar plots and heat maps were allowed to visualize the canonical pathways and DMRs. For DEGs, manually selected pathways with activation *Z* score >0.5 were presented as bar plot.

#### Targeted bisulfite sequencing

To validate the findings of the whole genome RRBS study, the isolated DNA samples were processed and analyzed using the Targeted Bisulfite Sequencing Service (Zymo Research, Irvine, CA). Assays were designed targeting CpG sites in the specified regions of interest (ROI) using primers created with Rosefinch, Zymo Research’s proprietary sodium bisulfite converted DNA-specific primer design tool. Following regions were targeted: (i) *Araf1* – (>mm10_dna range=chrX:20848583-20848893; strand = +); (ii) *Flt1* – (>mm10_dna range=chr5:147724930-147726782; strand = -); and (iii) *Vegfb* – (>mm10_dna range=chr19:6987203-6987964; strand = -). Design parameters were chosen such that PCR amplicons would ideally be bigger than 100 bp but smaller than 300 bp. In addition, primers were designed to avoid annealing to CpG sites at the region of interest to the maximum extent possible. Following primer validation, provided samples were bisulfite converted using the EZ DNA Methylation-Lightning™ Kit (Zymo Research, D5030) according to the manufacturer’s instructions. Multiplex amplification of all samples using ROI specific primer pairs and the Fluidigm Access Array™ System was performed according to the manufacturer’s instructions. The resulting amplicons were pooled for harvesting and subsequent barcoding according to the Fluidigm instrument’s guidelines. After barcoding, samples were pooled, purified (DNA Clean & Concentrator-5™), and prepared for massively parallel sequencing using a MiSeq V2 300bp Reagent Kit (Illumina)and paired-end sequencing protocol according to the manufacturer’s guidelines.

Sequence reads were identified using standard Illumina base-calling software and aligned back to the reference genome using Bismark.^54^ Paired-end alignment was used as default thus requiring both read 1 and read 2 be aligned within a certain distance, otherwise both read 1 and read 2 were discarded. Index files were constructed using the bismark_genome_preparation command and the entire reference genome. The --non_directional parameter was applied while running Bismark. All other parameters were set to default. Nucleotides in primers were trimmed off from amplicons during methylation calling. The methylation level of each sampled cytosine was estimated as the number of reads reporting a C, divided by the total number of reads reporting a C or T. Sequence data was demultiplexed and assessed for sample read coverage, mapping efficiency, unique CpG coverage, and bisulfite conversion rate. The methylation ratio (meth_ratio) is calculated by using methylated_CpG_count/total_CpG_count. The following number of high quality CpGs were included in the analysis: (i) *Araf1* = 19 CpGs; (ii) *Flt1* = 89 CpGs and (iii) *Vegfb* = 59 CpGs.

#### 5-mc and 5-hmC ELISA

Total genomic DNA was isolated from skin samples using the DNeasy Blood & Tissue Kit (Qiagen Cat # 69504) as per manufacturer’s instructions. For detecting global DNA methylation status (5-mC), the MethylFlash™ Methylated DNA Quantification Kit (Colorimetric; Epigentek, Cat # P-1030) was used. Additionally for detecting global DNA hydroxymethylation status, MethylFlash™ Global DNA Hydroxymethylation (5-hmC) ELISA Easy Kit (Colorimetric; Epigentek, Cat # P-1032) was used. A total of 100ng DNA was used for the assay. The absorbance was determined with the Synergy HTX multimode plate reader (Agilent) at 450 nm. To determine the specific methylation status of each DNA sample, the calculation of percentage of 5-mC or 5-hmC in total DNA was carried out using the following formula:

$$5\text{mC}\;\text{or}\;5\text{hmC}\%=\frac{\left(\text{sample}\;\text{OD}-\text{negative}\;\text{control}\;\text{OD}\right)}{\left(\text{slope}\;x\;S\right)}\;X\;100\%$$

Where S represents the amount of input DNA sample (ng).

#### Histology and immunohistochemistry (IHC)

Histology of skin was performed on skin tissue embedded in optimal cutting temperature (OCT) compound as reported.^55^ OCT-embedded tissue was cryosectioned (10 μm), fixed with cold acetone, blocked with 10% normal goat serum (NGS), and incubated with specific primary antibodies against anti-5-mC (#28692S, Cell Signaling Technology; 1:200 dilution), anti-5-hmC (#ab214728, abcam; 1:200 dilution), E-cadherin (#13–-1900, Invitrogen; 1:400 dilution), E-cadherin (#13-1900, Invitrogen; 1:400 dilution) CD31 (#550274, BD Pharmingen; 1:200 dilution), F4/80 (#MCA497R, Bio-Rad; 1:200 dilution); ARAF (#22129-1-AP, Proteintech Group, Inc.; 1:100 dilution) followed by appropriate fluorescence conjugated secondary antibodies (Alexa 568-tagged α-sheep, #A-21099, Invitrogen, 1:200 dilution; Alexa 568-tagged α-rat, # ab175476, Abcam, 1:200 dilution; Alexa 488-tagged α-rabbit, #A-11008, Invitrogen, 1:200 dilution). Images were collected and analyzed using a Zeiss Axio Scan Z1 microscope guided by Zen blue imaging software.^56^^,^^57^ IHC images were analyzed using the ImageJ software (NIH).^53^^,^^58^

### Quantification and statistical analysis

Statistical analysis and software used for RNA sequencing analysis, RRBS analysis, targeted bisulfite sequencing, 5-mc and 5-hmC ELISA, and IHC analysis is provided in the respective method sections, results and in the figure legends. Exact value of “n” and what it represents is presented in the legend of each figure. Definition of center, and dispersion and precision measures is provided in the legend of the respective figures.

## Supplemental information

Document S1. Figures S1–S5 and Tables S2 and S3

Table S1. Raw count and processed RNA sequencing data of skin of the mice of ESE group (n = 10) vs. the ground controls (n = 6), related to Figure 1 The analyzed raw data has been uploaded to GEO database under accession number GSE268755. Tab ST1A represents the information of individual RNA-seq libraries generated for the analysis. Tab ST1B represents raw counts of RNA seq data. Tab ST1C represents the analysis ready molecules as determined by IPA software. n represents number of animals.

Table S4. Table showing processed RRBS sequencing data of skin from the mice of responder, non-responder and the ground controls used in this study, related to Figure 3 The analyzed raw data has been uploaded to GEO database under accession number GSE268753. n represents number of animals. Tab ST4A represents the information of individual samples that were processed for RRBS analysis. Tab ST4B represents methylation difference between non-responder ESE cohort (n = 6) vs. ground control (n = 6). Tab ST4C represents methylation difference between responder ESE cohort (n = 4) vs. ground control (n = 6). n represents number of animals.

Table S5. Table showing RNA-seq analysis between ESE groups and ground control, related to Figure 6 Tab ST5A represents the information of individual RNA-seq libraries generated and information about differential expression (DE) analysis. Tab ST5B shows raw count and processed RNA sequencing data of skin of the mice from responder (n = 4), non-responder (n = 6) and ground controls (n = 6). The analyzed raw data has been uploaded to GEO database under accession number GSE268755. n represents number of animals.

Figure360. An author presentation of Figure 1
