# Mission SpaceX CRS-16 RRRM-1 space flight induced skin genomic plasticity via an epigenetic trigger

**DOI:** 10.1016/j.isci.2025.112683

**Published:** 2025-10-13

**Authors:** Kanhaiya Singh, Priyanka Verma, Rajneesh Srivastava, Yashika Rustagi, Manishekhar Kumar, Sumit S. Verma, Sujit Mohanty, Afshin Beheshti, Liz Warren, Chandan K. Sen

## Main text

(iScience *27*, 111382; December 20, 2024)

In the originally published version of this article, there were typographical errors the name of the space mission. “CRS-19” has been replaced with “CRS-16” throughout the manuscript.

Specific titles and a line of text in the original article:

• The title (in main and supplementary information) was originally “Mission SpaceX CRS-19 RRRM-1 space flight induced skin genomic plasticity via an epigenetic trigger”
• A line in the “Skin samples from mice” subsection of the STAR Methods originally read “These mice belonged to the SpaceX CRS-19 Rodent Research-8 mission [also known as Rodent Research Reference Mission-1 (RRRM-1)] were procured from ISS National Laboratory in collaboration with Dr. Liz Warren.”
• The title within the Figure 360 Figure Viewer was originally “Mission SpaceX CRS-19 RRRM-1 space flight induced skin genomic plasticity via an epigenetic trigger”

Revised, these now read:

• “Mission SpaceX CRS-16 RRRM-1 space flight induced skin genomic plasticity via an epigenetic trigger”
• “These mice belonged to the SpaceX CRS-16 Rodent Research-8 mission [also known as Rodent Research Reference Mission-1 (RRRM-1)] and were procured from ISS National Laboratory in collaboration with Dr. Liz Warren.”
• “Mission SpaceX CRS-16 RRRM-1 space flight induced skin genomic plasticity via an epigenetic trigger”

All authors agree to the correction and confirm that this does not impact the scientific conclusions of the paper.

