# Supplementary material for: Mission SpaceX CRS-16 RRRM-1 space flight induced skin genomic plasticity via an epigenetic trigger
Source: iScience. 2024 Nov 14;27(12):111382. doi: 10.1016/j.isci.2024.111382 (PMC11647166; doi:10.1016/j.isci.2024.111382)
Supplement: Document S1. Figures S1–S5 and Tables S2 and S3 [file mmc1.pdf]

iScience, Volume *27*

Supplemental information

Mission SpaceX CRS-16 RRRM-1 space flight induced skin genomic plasticity via an epigenetic trigger

Kanhaiya Singh, Priyanka Verma, Rajneesh Srivastava, Yashika Rustagi, Manishekhar Kumar, Sumit S. Verma, Sujit Mohanty, Afshin Beheshti, Liz Warren, and Chandan K. Sen

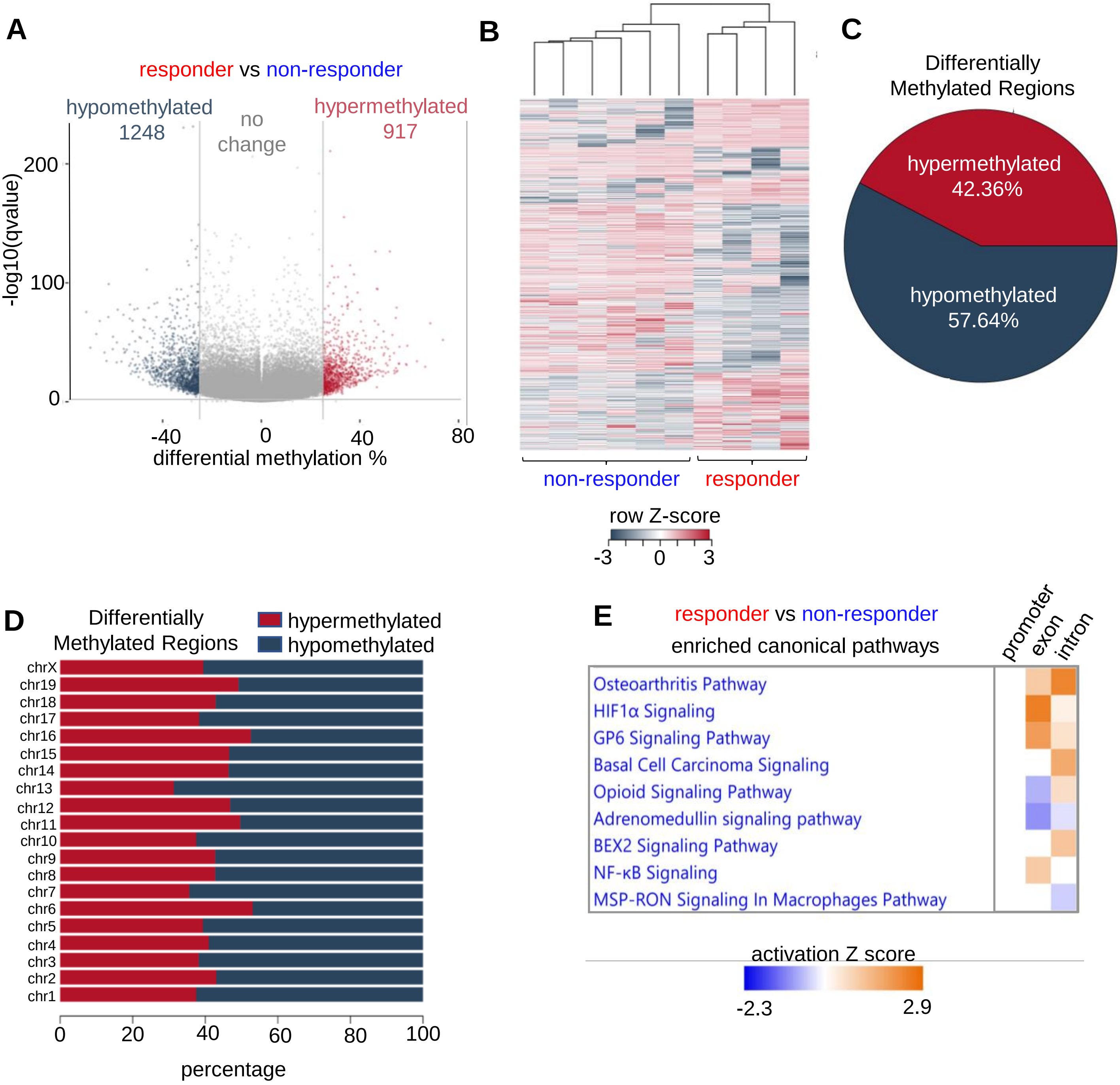

Figure S1: Hypomethylated DMRs were observed in responders when directly compared with non-responders, related to Figure 3. (**A**) Volcano plot of total methylated regions in responder ESE cohort (n=4) compared to non-responder ESE cohort (n=6). Out of total 100159 identified differentially methylated regions (DMRs), 2165 significant DMRs (differentially methylated with a q-value smaller than 0.01 and at least 25% difference) were identified. Out of 2165 significant DMRs, 1248 were hypomethylated in responder ESE cohort and 917 were hypermethylated. (**B**) Clustering heatmap of significant DMRs in responder ESE cohort (n=4) compared to non-responder ESE cohort (n=6). The 2165 significant DMRs were clustered and a heatmap was generated. (**C**) The pie chart shows the percentage of hyper and hypo methylated regions in the comparison. Out of 2165 significant DMRs, 1248 (57.64%) were hypomethylated in responder ESE cohort and 917 (42.36%) were hypermethylated. (**D**) The percentage of hypo and hyper methylated regions is plotted in a bar chart per chromosome in responder ESE cohort compared to non-responder ESE cohort. (**E**) Canonical pathways enriched by the DMRs annotated in exon, intron and promoter regions in responder ESE cohort (n=4) compared to non-responder ESE cohort (n=6) using comparison analysis function of Ingenuity Pathway Analysis (IPA). n represents number of animals.

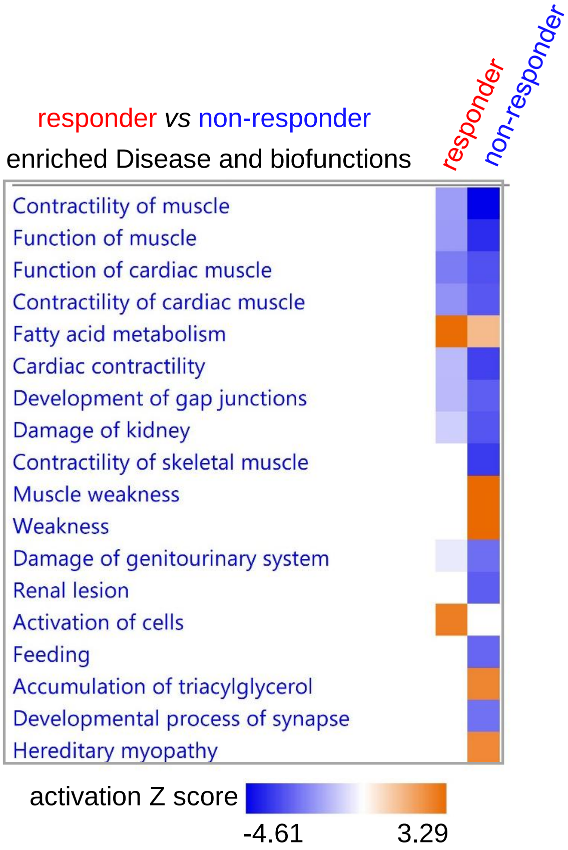

Figure S2: Non-responder mice were associated with loss of muscle function as compared to responders, related to Figure 3. Disease and biofunctions enriched by the DEGs in responder ESE cohort (n=4) compared to non-responder ESE cohort (n=6) using comparison analysis function of Ingenuity Pathway Analysis (IPA). Ground control group (n=6) was used as reference. n represents number of animals.

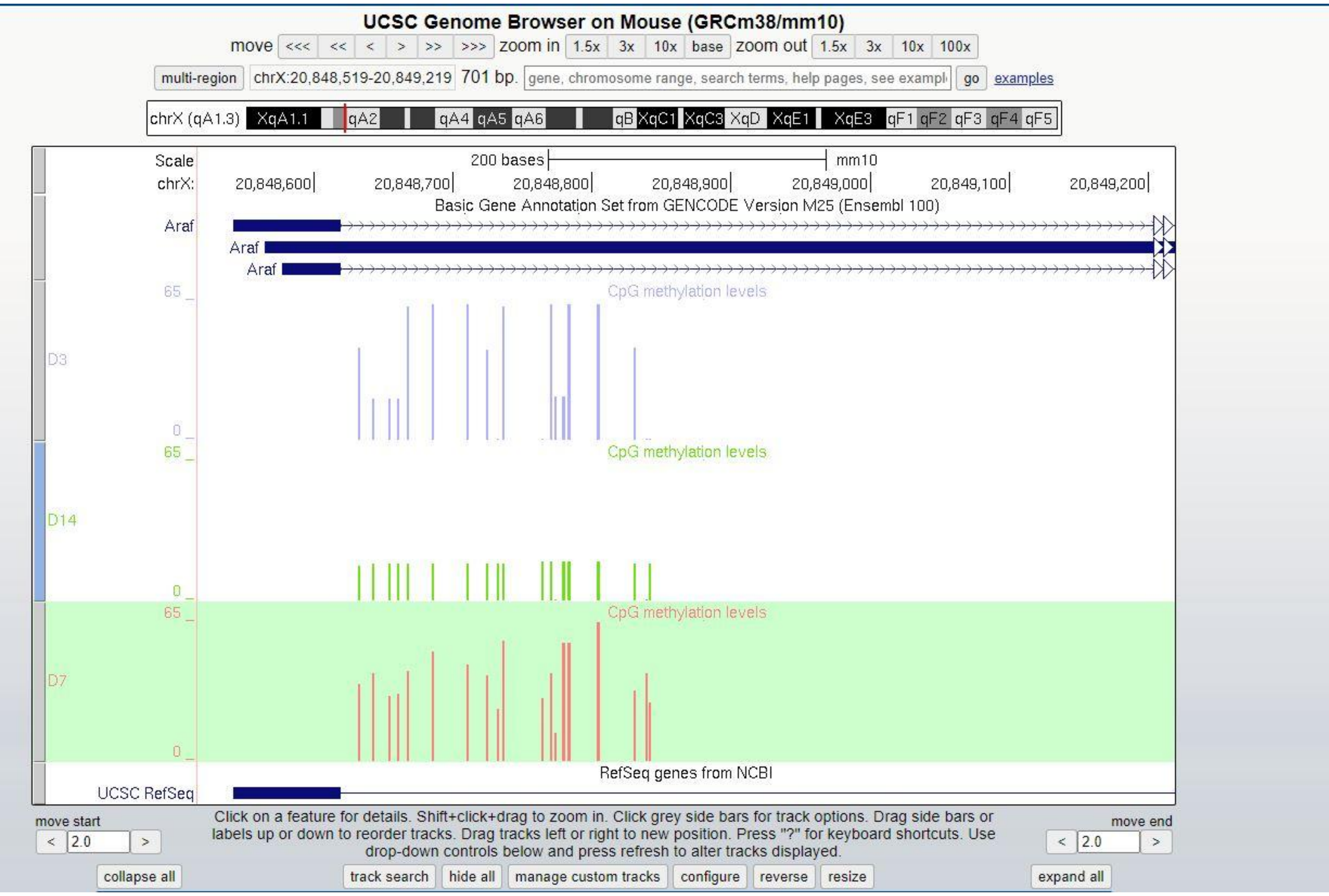

Figure S3: Full range of UCSC genome data for ARAF locus is presented, related to Figure 5. Full image of genome track shown in Figure 5A for the ARAF locus obtained using UCSC genome browser.

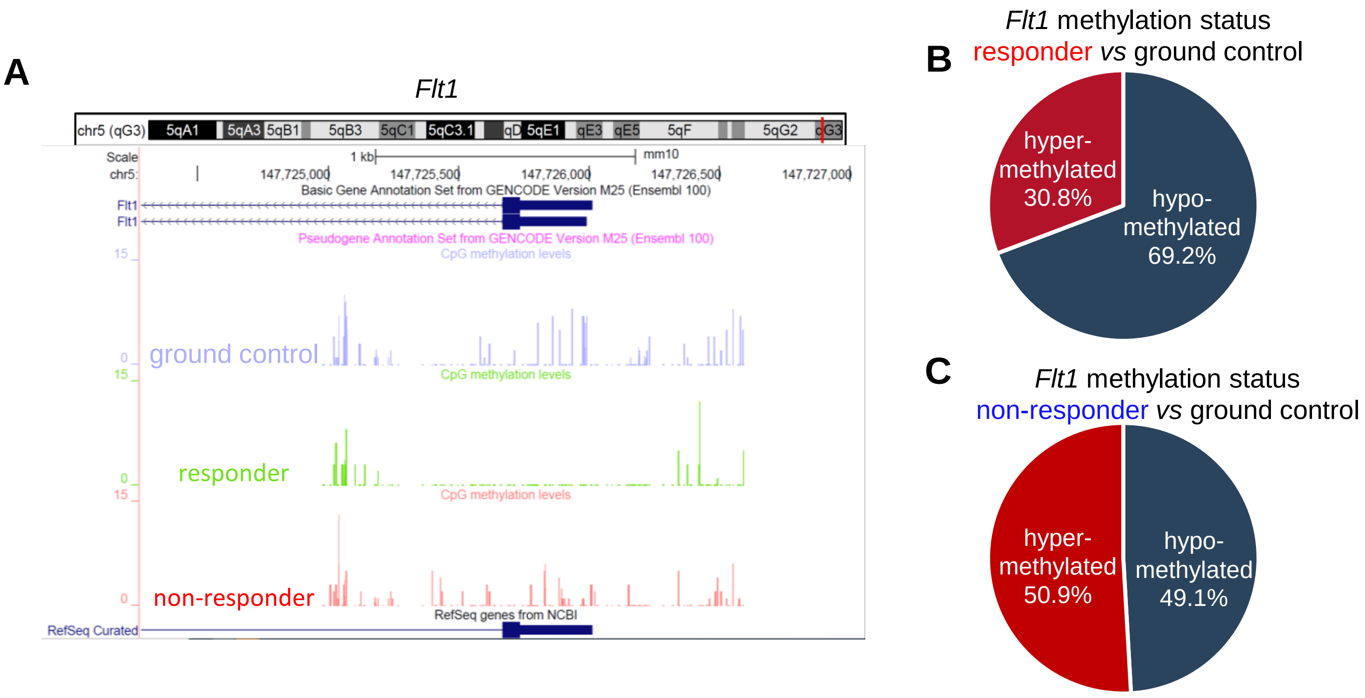

Figure S4: *Flt1* gene was hypomethylated in responder ESE cohort, related to Figure 6. (**A**) Targeted validation of *Flt1* gene promoter methylation status in ground control, responder ESE cohort and non-responder ESE cohort. Genome track showing the *Flt1* locus (top). Representative DNA methylation tracks (bottom) of show diminished levels of methylated CpGs in responder ESE skin as compared to non-responder ESE or control skin. (**B**) Venn diagram showing the percentage of hypomethylated (blue) and hypermethylated (red) CpGs in *Flt1* gene in responder ESE cohort (n=4) vs ground control (n=12) and (**C**) in non-responder ESE cohort (n=6) vs ground control (n=12). n represents number of animals.

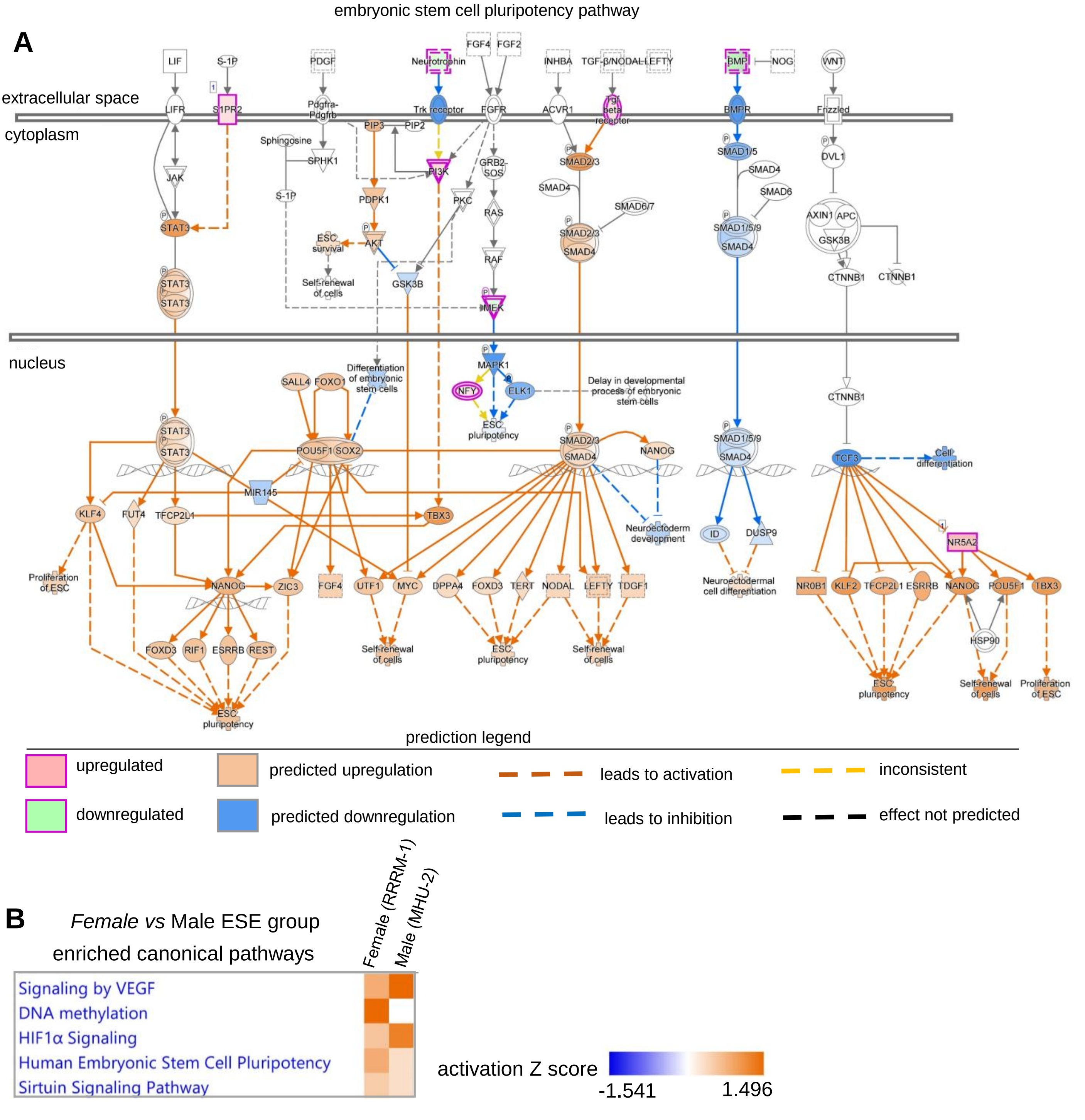

Figure S5: Upregulation of predicted biological functions such as ESC pluripotency, self-renewal of cells and proliferation of ESC were also observed in male mice similar to females, related to Figure 1. (A) Pathway generated by IPA showing the candidate genes involved in the embryonic stem cell (ESC) pluripotency pathway enriched in male mice from JAXA MHU-2 mission. The log2FC was obtained using NASA Open Science for Life in Space repository (GeneLab ID: GLDS-239; DOI: 10.26030/s7k9-7958). Among the biofunctions that were predicted to be upregulated in the skin samples of male ESE group were: ESC pluripotency, self renewal of cells and proliferation of ESC. In the JAXA MHU-2 mission, twelve 16-18 week old male C57BL/6J mice were singly housed in the JAXA Habitat Cage Units (HCUs) on the ISS for 30 days. Three flight mice were housed in microgravity and received standard JAXA chow. Mice were returned live and euthanized and dissected <1 day after splashdown. Ground controls (n=3) were asynchronous, housed in HCUs and received standard chow. **(B)** Canonical pathway enriched by the DEGs in female ESE cohort (RRRM-1) compared to male ESE cohort (MHU-2) using comparison analysis function of IPA. n represents number of animals.

Symbol	Entrez Gene Name	Ensembl	Expr Log Ratio	IPA Expected Trend	Location	Type(s)
ACVR1	activin A receptor type 1	ENSMUSG00000026836	0.515	Up	Plasma Membrane	kinase
BMP4	bone morphogenetic protein 4	ENSMUSG00000021835	-1.053	Up	Extracellular Space	growth factor
CTNNB1	catenin beta 1	ENSMUSG00000006932	0.421	Up	Nucleus	transcription regulator
FZD9	frizzled class receptor 9	ENSMUSG00000049551	-2.048	Up	Plasma Membrane	G-protein coupled receptor
MAP2K3	mitogen-activated protein kinase kinase 3	ENSMUSG00000018932	-0.59	Up	Cytoplasm	kinase
NOG	noggin	ENSMUSG00000048616	-1.436	Down	Extracellular Space	growth factor
NR5A2	nuclear receptor subfamily 5 group A member 2	ENSMUSG00000026398	3.47	Up	Nucleus	ligand-dependent nuclear receptor
NTRK3	neurotrophic receptor tyrosine kinase 3	ENSMUSG00000059146	2.565	Up	Plasma Membrane	kinase
PDGFA	platelet derived growth factor subunit A	ENSMUSG00000025856	-0.357	Up	Extracellular Space	growth factor
PRKCQ	protein kinase C theta	ENSMUSG00000026778	-1.511	Up	Cytoplasm	kinase
PRKD1	protein kinase D1	ENSMUSG00000002688	1.751	Up	Cytoplasm	kinase
TERT	telomerase reverse transcriptase	ENSMUSG00000021611	1.082	Up	Nucleus	enzyme
WNT6	Wnt family member 6	ENSMUSG00000033227	0.977	Up	Extracellular Space	other

Table S2: **Genes belonging from human embryonic stem cell (ESC) pluripotency pathway that were differentially expressed in mouse skin on exposure to space environment (ESE), related to Figure 1**. It was interesting to note that 8 out of 13 followed the expected trend in the literature as calculated by IPA. Hence ESC pathway was predicted to be upregulated in response to ESE.

Sample ID	ISS groups	%5methyl cytosine	Groups Inferred based on 5mC ELISA
HGC-ISS-01	Habitat Ground Control	0.438474026	Ground control
HGC-ISS-02	Habitat Ground Control	0.398376623	Ground control
HGC-ISS-03	Habitat Ground Control	0.432954545	Ground control
HGC-ISS-04	Habitat Ground Control	0.487824675	Ground control
HGC-ISS-05	Habitat Ground Control	0.473051948	Ground control
HGC-ISS-06	Habitat Ground Control	0.46737013	Ground control
HGC-ISS-07	Habitat Ground Control	0.325324675	Ground control
HGC-ISS-08	Habitat Ground Control	0.388474026	Ground control
HGC-ISS-09	Habitat Ground Control	0.570292208	Ground control
HGC-ISS-10	Habitat Ground Control	0.57224026	Ground control
HC-LAR-01	Habitat Ground Control	0.964285714	Ground control
HC-LAR-02	Habitat Ground Control	0.705681818	Ground control
HC-LAR-05	Habitat Ground Control	0.710227273	Ground control
HC-LAR-06	Habitat Ground Control	0.693993506	Ground control
HC-LAR-13	Habitat Ground Control	0.635064935	Ground control
HC-LAR-14	Habitat Ground Control	0.682954545	Ground control
HC-LAR-17	Habitat Ground Control	0.716883117	Ground control
HC-LAR-18	Habitat Ground Control	0.875324675	Ground control
FL-ISS-01	Euthanized in space	0.695941558	non-responder
FL-ISS-03	Euthanized in space	0.37012987	non-responder
FL-ISS-04	Euthanized in space	0.69512987	non-responder
FL-ISS-05	Euthanized in space	0.602922078	non-responder
FL-ISS-06	Euthanized in space	0.438961039	non-responder
FL-ISS-08	Euthanized in space	0.831818182	non-responder
FL-ISS-09	Euthanized in space	0.73474026	non-responder
FL-ISS-10	Euthanized in space	0.65	non-responder
FL-LAR-02	Euthanized post-landing	0.568506494	non-responder
FL-LAR-05	Euthanized post-landing	0.526136364	non-responder
FL-LAR-06	Euthanized post-landing	0.583603896	non-responder
FL-LAR-13	Euthanized post-landing	0.405519481	non-responder
FL-LAR-17	Euthanized post-landing	0.619805195	non-responder
FL-ISS-02	Euthanized in space	0.012175325	responder
FL-ISS-07	Euthanized in space	0.022402597	responder
FL-LAR-01	Euthanized post-landing	0.008441558	responder
FL-LAR-18	Euthanized post-landing	0.015422078	responder

Table S3: **Table showing 5-methylcytosine (5-mC) levels in skin tissue studied in mice were flown in an established Rodent Habitat on the ISS (n=17) and ground-based mice (ground controls; n=18) maintained under similar housing conditions, related to Figure 2**. Exposure to ESE on DNA global methylation identified two distinct groups – non-responders and responders exclusively based on global methylation levels represented as percentage of 5-mC. n represents number of animals.
